# Molybdenum, Vanadium, and Tungsten-Based Catalysts for Sustainable (ep)Oxidation

**DOI:** 10.3390/molecules27186011

**Published:** 2022-09-15

**Authors:** Jana Pisk, Dominique Agustin

**Affiliations:** 1 LCC-CNRS, Université de Toulouse, CNRS, UPS, CEDEX 4, F-31077 Toulouse, France; 2 Department of Chemistry, Institut Universitaire de Technologie Paul Sabatier, University of Toulouse, Av. G. Pompidou, BP20258, CEDEX, F-81104 Castres, France; 3 Department of Chemistry, Faculty of Science, University of Zagreb, Horvatovac 102a, 10000 Zagreb, Croatia

**Keywords:** molybdenum, vanadium, tungsten, polyoxometalates, catalysis, oxidation, green methods, supported catalysts, biomass valorization

## Abstract

This article gives an overview of the research activity of the LAC2 team at LCC developed at Castres in the field of sustainable chemistry with an emphasis on the collaboration with a research team from the University of Zagreb, Faculty of Science, Croatia. The work is situated within the context of sustainable chemistry for the development of catalytic processes. Those processes imply molecular complexes containing oxido-molybdenum, -vanadium, -tungsten or simple polyoxometalates (POMs) as catalysts for organic solvent-free epoxidation. The studies considered first the influence of the nature of complexes (and related ligands) on the reactivity (assessing mechanisms through DFT calculations) with model substrates. From those model processes, the work has been enlarged to the valorization of biomass resources. A part concerns the activity on vanadium chemistry and the final part concerns the use of POMs as catalysts, from molecular to grafted catalysts, (ep)oxidizing substrates from fossil and biomass resources.

## 1. Introduction

Among the several challenges that the chemical industry has to face in the future, the most visible is to diminish/erase its negative image, soiled for years by several unfortunate contaminations issues, hazards being mainly bad handling procedures and storage mistakes (ammonium nitrate explosions, chemical leaks from tankers, burning of chemicals) [1]. For a century, the market of organic molecules has mainly been based on relatively cheap and available fossil resources. The “cheap” version of those raw sources is diminishing and industries have to face some geopolitical issues, increasing their prices. Maintaining the production of such organic molecules, with a relative low price/cost, obliges academic/industrial chemists to consider the development of new sources. New fossil sources can still be discovered for several years (ca. 50–100 according to Association for the Study of Peak Oil-ASPO) but with low accessibility [2,3]. The use of fossil sources being one cause of global warming, the quest toward renewable and sustainable sources seems more than urgent. In the current time, there is high interest in the carbon footprints of all processes/products for public policies but also for energy- and matter-saving processes. This was among the reasons that academic and industrials scientists began to think about solutions for a “better chemistry”. Those ideas have been gathered in 12 principles in the beginning of 1990 called “Green Chemistry” [4]. This concept points out the urgent need for new processes that are more sustainable and less energy demanding. Presented solutions recommend using cleaner and safer processes to replace the actual chemical processes where hazards might more frequently occur [5]. All of those ideas are a straight line to solutions anticipating fossil depletion.

To save energy in a chemical process, one point is to diminish the activation energy of a chemical reaction. For this, catalytic processes, among the 12 principles pointed out by Anastas and Warner, are one relevant solution [6]. In addition to reactions in which the catalyst diminishes the activation energy barrier, making the chemical reaction faster and preferably more selective, the process should become cleaner by replacing, diminishing, or eliminating organic solvents (sometimes toxic and often from fossil sources) and finding new, renewable sources [7]. Several research groups developed new answers with one or more of those solutions. In the presented research, we have followed Green Chemistry principles, i.e., namely *catalysis*, *catalysts recovery* through grafting, *organic solvent-free* processes, and *biomass valorization*. Those objectives are realized in the LCC research group in close collaboration with international research groups, especially with the one from Croatia. All that is presented herein corresponds to the work developed by Castres for several years, within the frame of the LCC research group devoted to catalysis. Within the numerous possible simple chemical transformations, the work developed herein focused on oxidation reactions, i.e., olefin epoxidation and alcohol oxidations.

Oxidation processes are at the origin of the formation of numerous molecules present in nature. From a fundamental point of view, studying those processes helps to understand the formation of those compounds. From an applicative point of view, chemists tend to mimic faster natural oxidation processes in order to obtain in abundant quantities (and preferably with a decent price) molecules present in nature (but often in too small a quantity considering commercial application) [8] or to create new molecules for several other purposes (often pharmaceutical). The advantage of oxidation protocols is to be under air, in agreement with some principles of Green Chemistry (simple process). The impact of such reactions is huge since oxidation represents a big part of industrial chemical transformation. The pharmaceutical industry [9,10,11], polymer industry [12,13], as well as the flavor and fragrance industries [14,15] need simple building blocks, and starting reagents have to be easily accessible. For example, the most known efficient synthetic processes to perform olefin epoxidation or alcohol oxidation use non-green conditions. The use of toxic inorganic oxidants in a stoichiometric amount, strong acids and/or organic solvents represent the non-green area that has to be replaced in light of the previously cited Green Chemistry principles and safety regulations [16,17].

Some existing processes are found to be cleaner, including metal complexes and/or metal oxides as catalysts, with the use of a cleaner oxidant as H_2_O_2_ [18], TBHP [19], or O_2_ [20]. Among efficient metals, we focused on transition metals with low toxicity, i.e., Mo, W, and V. Most of those metal-catalyzed processes used organic solvents and, in the case of epoxidation especially, dichloroethane (DCE) has been found to the most efficient. The replacement of DCE and extension to any organic solvent is an interesting challenge that has been discussed in the context of the industrial sector [21,22,23].

“*No solvent is the best solvent*” is the motto of the catalyzed processes presented herein. For this, we have separated the work into several aspects, that dealing with molybdenum, tungsten, and vanadium coordination complexes containing mainly two types of tridentate ligands, including some mechanistic studies as well as valorization of biomass. The second aspect will focus on the use of commercial polyoxometalates as oxidation catalysts under organic solvent-free protocols, using organic salts and grafted salts, for simple model studies and on applied processes toward the synthesis of useful species or the use of biomass substrates.

## 2. Tridentate Ligands and Related Complexes

This section considers coordination complexes containing ONO or ONS coordination sphere tridentate ligands, some backbones derived from the salicylideneaminophenol (SAP), and those bearing hydrazone moieties. The results are collected according to the nature of the metal and the ligand in the case of fundamental studies and valorization is added in an extra sub-chapter.

### 2.1. Molybdenum and Tungsten Complexes

#### 2.1.1. From SAP Ligands and Derivatives: Mechanistic Study

*Methodological Approach and Model Study with Cyclooctene*

Among the metal-containing species exhibiting interesting catalytic activity, molybdenum was quite active and deeply used. Coordination complexes bearing tridentate ligands attracted our attention. Indeed, Mo is known to be at the core of several industrial processes, but conditions were not as green as they could be [24,25]. The work engaged herein started from the chemistry of [MoO_2_L] complexes with tridentate ligands, accessible complexes for sustainable chemistry. The studied tridentate H_2_L ligand (H_2_SAP) is obtained as the “Schiff base” formation by condensation of 1,2-aminophenol and salicylaldehyde. The related molybdenum complex is obtained starting from one precursor, [MoO_2_(acac)_2_] (acac = acetylacetonate), reacting in a stoichiometric amount with H_2_L ligand. [MoO_2_(SAP)(EtOH)] complex was previously described, and its catalytic activity toward epoxide was shown in organic media. In several articles from Sobczak and Ziółkowski [26,27], a good catalytic activity of the [MoO_2_(SAP)(EtOH)] was announced and placed the complex as a very promising catalyst. In the frame of a sustainable process, the reaction could be improved since the reaction was performed in the presence of a halogenated solvent (bromobenzene) and needed TBHP in decane as an oxidant, i.e., the presence of two organic solvents, one being halogenated.

**Figure 1:**
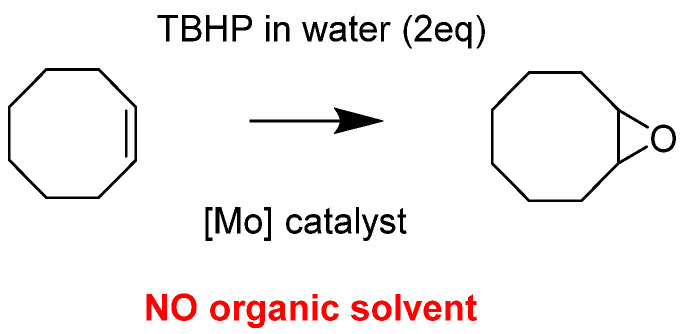
Organic solvent-free epoxidation of cyclo-octene.

The idea developed herein has been to test the reactivity of the “MoO_2_(SAP)” complex and derivatives as catalysts in a more sustainable way. Thus, catalytic experiments were done in absence of any organic solvent, using TBHP in water as an oxidant and no organic solvent addition than the model substrate itself within the reaction media, i.e., cyclooctene (Figure 1). Water (from TBHP) was not a solvent in the process since it was observed that a biphasic system occurred with the presence of the catalyst within the organic phase (phase containing the substrate itself). As previously mentioned, the catalytic activity of those species was interesting and a beginning of mechanism postulation proposed an equilibrium between [MoO_2_L]_2_ and [MoO_2_L(ROH)] within the reaction media, being the starting base of the postulated mechanism with a potential pentaco-ordinate species [MoO_2_(L)] calculated by DFT and explained later.

*Preliminary Studies toward the Best Backbone and Mechanistic Study*

**Figure 2:**
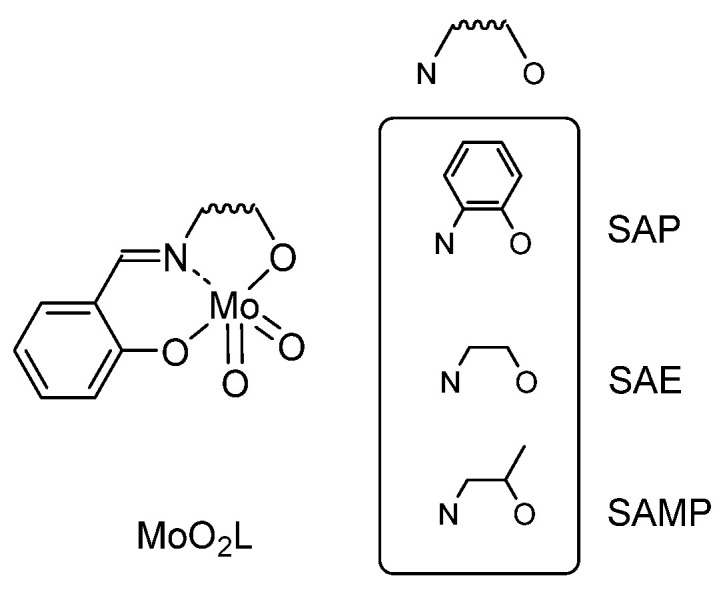
Preliminary studied MoO_2_L structures.

Two different ligands were synthesized using salicylaldehyde and 1,2 aminoethanol (leading to H_2_SAE ligand) or 1,2 aminophenol (leading to H_2_SAP ligand), Figure 2 [28]. Molecular structures of [MoO_2_(SAP)(EtOH)] and a polymorphic form of [MoO_2_(SAE)(SAEH_2_)] [29] were determined by X-ray diffraction. The “MoO_2_L” species have been studied in DFT according to the previously mentioned mechanism [26,27] and it was shown that both [MoO_2_(L)]_2_ (L = SAP, SAE) complexes needed less energy to be converted into the pentaco-ordinate mononuclear complex [MoO_2_L] than their ethanol-stabilized congeners [MoO_2_L(EtOH)] [28].

**Figure 3:**
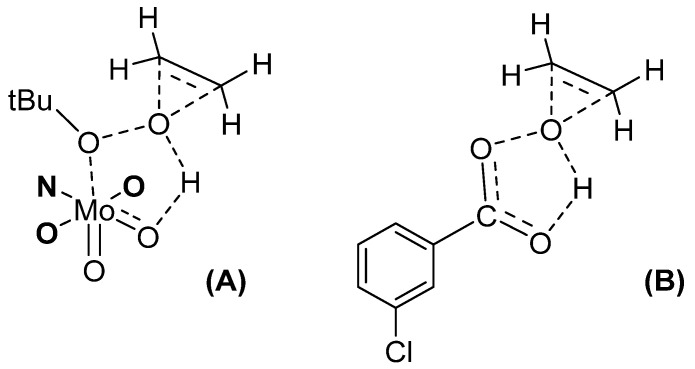
The transition state (TS) of olefin epoxidation using TBHP and MoO_2_(SAP) catalyst (**A**) vs. the *m*-CPBA postulated mechanism (**B**). For clarity of the drawing, the H_2_SAP ligand was limited to the ONO coordination sphere.

The catalytic activity of [MoO_2_L]_2_ and [MoO_2_L(MeOH)] complexes (L = SAE, SAMP, SAP) was studied through cyclo-octene epoxidation [30]. The nature of the ligand helped the system to be more active. Indeed, “MoO_2_(SAP)” based complexes were stable, but the SAE and SAMP ligands from complexes were hydrolyzed during the catalytic process. An induction period observed with the [MoO_2_L(MeOH)] species and not [MoO_2_L]_2_ confirmed that more energy was needed to deco-ordinate the monomer stabilized by a solvent than the dimer into the pentacoordinate active species, confirmed by the DFT calculations. A direct relationship could be established between the catalyst stability and selectivity. From the experimental work with those complexes, one DFT-calculated pathway fitted to experimental observations, showing a new type of TBHP activation, corresponding to the Bartlett postulation done in the Prizhaev reaction, i.e., when an olefin does react with a peroxyacid as *m*-CPBA [31]. The transition state (TS) corresponds to a loose coordination of TBHP to the Mo together with an H-bonding with an oxido moiety linked to the Mo center. From this, the oxygen atom from the TBHP linked to the hydrogen can be transferred to the olefin (as seen in Figure 3A). Although [MoO_2_(acac)_2_] is an active catalyst, the advantage of [MoO_2_(SAP)] derivatives lie in their strong stability.

*Ligand Tuning and Optimization*

**Figure 4:**
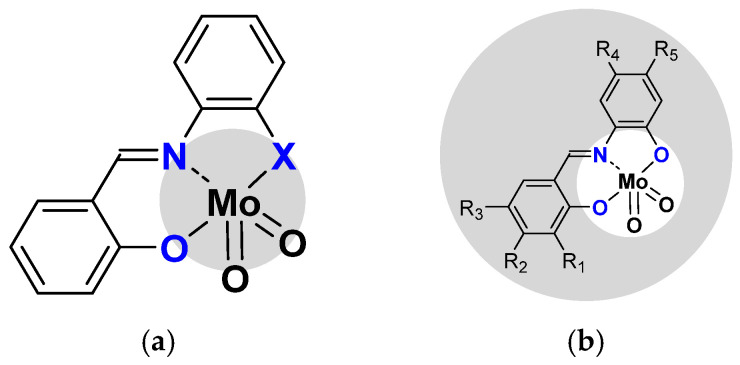
(**a**) Modification of coordination sphere X = O vs. X = S (**b**) Modification of the second sphere on R_1_ to R_5_ positions (specified in the text and in Table 1).

From this mechanism, optimization of the process was pursued using a slight modification of the ligand. The first change has been on the coordination sphere, comparing ONO with the ONS coordination sphere [32]. The [MoO_2_(SATP)] complex (ONS coordination sphere) was more active than [MoO_2_(SAP)] one (ONO coordination sphere) and activity was confirmed with DFT calculations (Figure 4a).

The modifications of the pending organic functions surrounding the H_2_SAP ligand (second co-ordination sphere) were performed to evaluate their effects (according to nature and/or position) on catalytic properties (Figure 4b). Additional DFT calculations could help to refine the proposed theoretical model. Thus, different substituents have been added and activity was compared toward cyclo-octene, as shown in Table 1.

The addition of functional groups (R_2_ = diethylamino and/or R_5_ = nitro groups known for push-pull effects and modifications of NLO properties) at specific positions on the SAP backbone led to the comparison of four different molecules based on the presence or absence of both substituents [33]. The electronic effect of substituents on ligand might influence the electronic density of the molybdenum atom and subsequently the catalytic activity of the MoO_2_L complexes. Indeed, compared to [MoO_2_(SAP)] where no substitution occurred, the presence of the NO_2_ electron withdrawing group on the ligand increased the conversion, while the presence of electron-donating ligand NEt_2_ gave a reverse effect. The presence of both groups together inhibited their effects and the catalytic activity of the [MoO_2_L] complex was similar to [MoO_2_(SAP)]. Those experimental data were confirmed by DFT calculations with enthalpies values of the transition state (TS) in agreement with activity. A low TS (according to Figure 3) value was observed for highly active processes and vice-versa.

The presence of the OH group on the salicylic part of the ligand was also interesting to study. Thus, the effect of the presence of one pending OH function in *ortho* (R_1_), *meta* (R_2_), or *para* (R_3_) position to the phenolic oxygen atom linked to Mo on catalytic activity has been studied with cyclo-octene as a model substrate and compared to [MoO_2_(SAP)] moiety. The OH position strongly influences the activity toward cyclooctene with activity vs. [MoO_2_(SAP)] very high, slightly higher, and identical when OH lie in *ortho*, *para*, and *meta* positions, respectively [34]. The behavior was confirmed through DFT calculations with similar trends.

Other groups (OMe on *ortho* position (R_1_) as for the OH and Me on the aminoalcohol part of the ligand (R_4_ and R_5_)) have been added on SAP ligand and the corresponding [MoO_2_L(MeOH)] species (obtained through mechanochemistry and a solventless protocol, LAG) have been tested on different substrates [35]. With cyclo-octene, results showed that the presence of the methoxy group was the main factor toward a better activity. The methyl substituents in R_4_ and R_5_ positions did not have a noticeable effect.

#### 2.1.2. ONS, ONO Mo-Enlarging the Scope of Complexes through Collaboration

From the knowledge of [MoO_2_L] species to act as catalysts under organic solvent-free conditions, a collaboration has been established with Croatia in 2010. All catalytic results have been separated according to the nature of the ligands around the molybdenum. Carbazones, hydrazones, and thiosemicarbazones ligands have been developed and presented according to the nature of the hydrazide used. For all the species with such geometries, we emphasized the TON (Turn Over Number) and TOF (Turn Over Frequency) parameters of the catalytic processes. TON is defined as the number of substrates transformed per unit of catalyst at the end of the studied time, i.e., the number of cycles per catalyst. TOF corresponds to the number of substrates transformed per unit of catalyst in time intervals all along a reaction. This shows how the reaction can start fast or not, according to the catalyst.

*Pyridoxal Fragment within the Ligand*

*ONS coordination sphere–thiosemicarbazones*

**Figure 5:**
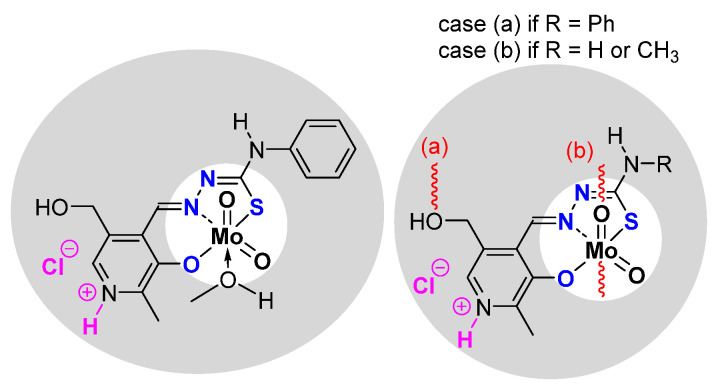
Monomeric and polymeric species studied containing the pyridoxal fragment in the thosemicarbazonato ligand. Species are neutral but can be also charged (pink part).

The synthesis of the thiosemicarbazonato dioxomolybdenum complexes based on pyridoxal ligand has been developed in Zagreb [36]. Pyridoxal possesses a free CH_2_OH pending function on a pyridine ring. On the other side of the ligand, thiosemicarbazide precursors possess different possible R terminal groups (R = H, Me, Ph). (Figure 5) Depending on the pyridoxal protonation; the formed molybdenum species can be neutral “[MoO_2_L]” or charged “[MoO_2_LH]Cl”. According to the presence (resp. absence) of donor molecules (for instance methanol), species can be stabilized as the monomer (resp. polymer) with polymerization mode influenced by the nature of R.

Those species have been tested as catalysts and results (Table 2) indicated that the nature of R, catalytic ratio, charge (neutral/anionic) of the complex, and monomeric/polymeric form are all factors influencing the catalytic results.

The most efficient catalyst under organic solvent-free conditions is [MoO_2_L(MeOH)], i.e., the neutral complex (with R = Ph) stabilized by a molecule of methanol, while corresponding polymer [MoO_2_L]_n_ (stabilized through the CH_2_OH pending function of a second MoO_2_L complex) is the less active. The studies showed that the addition of MeOH in high quantity inhibited the activity of the catalyst at the same level as the corresponding polymer itself. This study exhibited the role of methanol and its strong donation effect. All the equilibria within the process are in favor of the previously postulated mechanism in which the active species is the pentaco-ordinate complex [MoO_2_L] [37].

The charged complexes could be monomers or polymers as observed for the neutralcharges. The activity was lower than the neutral charges but the lower activity of the charged species corresponding to the most active neutral compound was more pronounced with monomers than for polymers [38].

*ONO coordination sphere–hydrazones*

**Figure 6:**
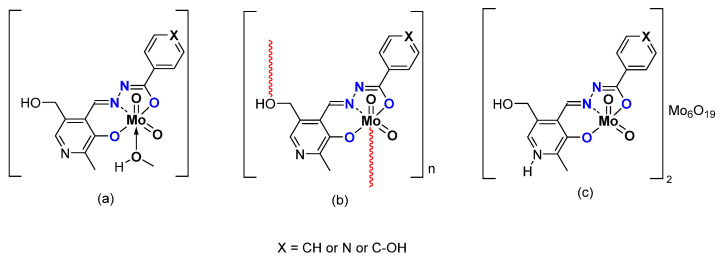
Schematic representation of neutral monomeric (**a**) polymeric (**b**) and charged mixed hybrid (**c**) species studied containing the pyridoxal fragment in the hydrazonato ligand.

Other pyridoxal containing Mo complexes have been studied, using ligands bearing a hydrazonato function and a ONO coordination sphere (Figure 6). The difference lies in the X, being phenyl (X = CH), pyridine (X = N) or a phenol group (X = C-OH). As for the ONS, monomeric [MoO_2_L(MeOH)] or polymeric [MoO_2_L]_n_ species were obtained. Interestingly, mixed hybrid compounds could be obtained, formally composed by two cationic coordination complex moieties [MoO_2_(LH)]^+^ and one Lindqvist polyanion [Mo_6_O_19_]^2−^ [39]. Those species (Figure 6) were tested as catalysts under the same experimental conditions (Table 3). Conversion of cyclooctene depends on the nature of the species, i.e., monomer, polymer, or hybrid. Considering monomers vs. polymers, the monomeric complex [MoO_2_L(MeOH)]-with the highest conversion after 6 h (X = C-H)- has the lowest in a polymeric state. As for the ONS species, the monomeric species bear higher TOF, i.e., faster activation. The species containing POMs are more active with a higher conversion (they contain two potential active substances) but the higher selectivity is due to the [MoO_2_L] species.

*From Mo to W Complexes with Salicylaldehyde Part: Some Experimental Features*

**Figure 7:**
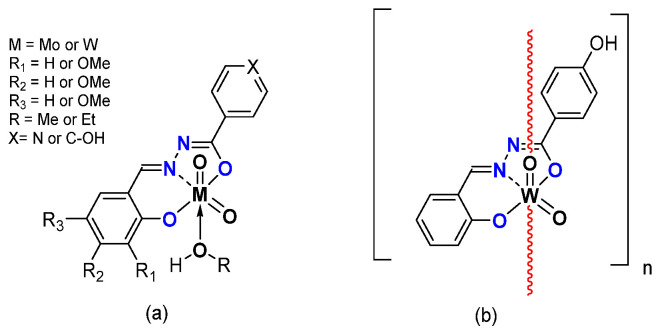
Schematic representation of neutral monomeric (**a**) and polymeric (**b**) Mo and Wspecies studied containing the salicylaldehydato fragment in the hydrazonato ligand.

Replacing the pyridoxal moiety by differently substituted salicylaldehydes led to monomeric [MO_2_L(EtOH)] (M = Mo, W) and polymeric [WO_2_L]_n_ species [40] (Figure 7). The catalytic activity of molybdenum complexes was quite high (Table 4), with conversion up to 90% and selectivity quite low compared to the complexes containing pyridoxal moieties. Tungsten-related complexes exhibited very low activity under the same experimental conditions. The oxidant TBHP in water did not seem to be the right oxidant to achieve good conversion and good selectivity.

The quest toward better experimental conditions was done with new ligands keeping the salicyladehyde moiety but adding isonicotinoyl hydrazines (X = C-OH), leading to [WO_2_L(MeOH)] and [WO_2_L(EtOH)] complexes [41].

Those species were tested under several experimental conditions. The reaction was faster using acetonitrile with TBHP in water as an oxidant but selectivity was worse than without acetonitrile. Reaction performed with TBHP in decane exhibited equivalent conversion than with TBHP in water but with higher selectivity, exhibiting the role of water. The effect of the coordinating alcohol was noticed, with a better conversion with EtOH, certainly due to a looser interaction with the W and a faster activation into a pentaco-ordinated species.

**Figure 8:**
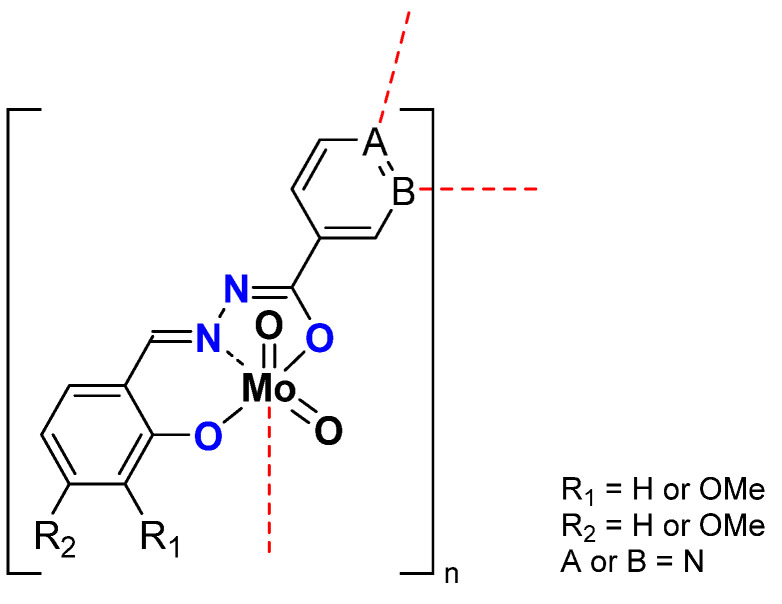
Schematic representation of neutral polymeric Mo species studied containing the nicotinoyl hydrazonato ligand.

In the case of nicotinoyl hydrazides and molybdenum complexes (Figure 8), it was shown that the oligomerization rate has an influence on the reactivity. Indeed, while all compounds existed in polymeric form (with high nuclearity) specific experimental conditions could lead to the isolation of tetranuclear scaffolds that appeared to be highly active, more than the corresponding polymer. (Table 5) It exhibited that the mechanism suggesting the formation of pentacoordinate species was relevant and easier with oligomers than for polymers [42]. In this case, it was also shown that TBHP in decane was more efficient [43].

**Figure 9:**
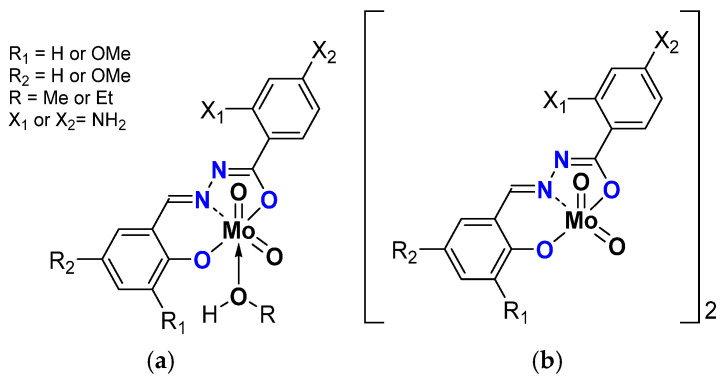
Schematic representation of neutral monomeric (**a**) and polymeric (**b**) Mo species studied containing the salicylaldehydato fragment in the hydrazonato ligand.

Oligomerization was emphasized with compounds having 4-aminobenzhydrazone ligands (Figure 9) [44]. Those species existed as monomer [MoO_2_L(ROH)] and dimer [MoO_2_L]_2_ due to the ligand bearing NH_2_ substituent coordinating the Mo of a neighboring molecule. The dimers reacted faster than the monomers, another proof of the postulated mechanism (Table 6). The comparison with the complexes bearing 2-aminobenzydrazones showed that the position of the NH_2_ is important within the stabilization of the transition state since those species were even more active. This has also been assessed through DFT calculations [45].

As expected, reactivity in TBHP in decane was faster. Different induction periods were observed between monomers and polymers and the substitution on the benzaldehydic part of the ligand seemed to have an influence.

**Figure 10:**
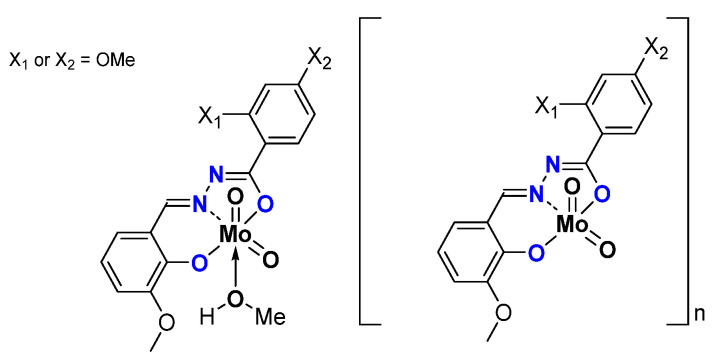
Schematic representation of neutral oligomeric and polymeric species studied containing the hydrazonato ligand.

Replacing -NH_2_ by -OH led to a series of monomeric and oligomeric MoO_2_L complexes (Figure 10) tested as catalysts using TBHP as an oxidant in water (W) or in decane (D) and no other added solvent [46]. Results are compiled in Table 7. Very good results are obtained with such structures, exhibiting the role of OH on both aromatic rings.

**Figure 11:**
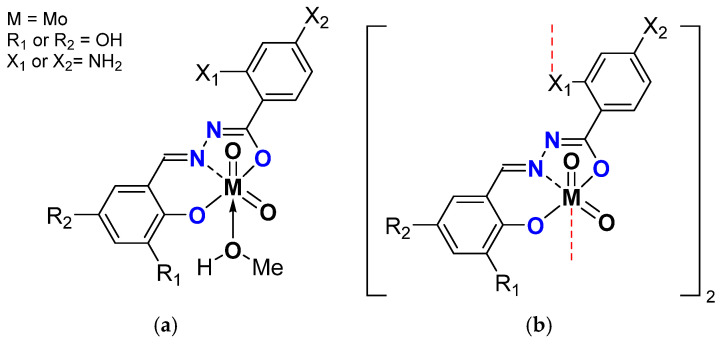
Schematic representation of neutral monomeric (**a**) and polymeric (**b**) Mo species studied containing the salicylaldehydato fragment in the hydrazonato ligand.

Very recently, another variation was done, replacing the MeO on the benzaldehydic part with OH on the *ortho* and *para* position and having the 2- or 4- NH_2_ benzydrazide (Figure 11) [47].

With those compounds, catalysts tests with cyclo-octene have been performed using three types of oxidants (Table 8), TBHP in water (W), TBHP in decane (D), and H_2_O_2_ in water (Table 8). As seen previously seen, TBHP in decane as oxidant gives the best results but TBHP in water gives relatively good results and is greener. With H_2_O_2_, the protocol was tested herein for the first time and the reaction was shown to be quite slow. A mechanistic study with H_2_O_2_ as oxidant showed that the less energetic mechanism is similar to the mechanism with TBHP.

### 2.2. Extension to High-Valued Species

#### 2.2.1. Other Olefins

**Figure 12:**
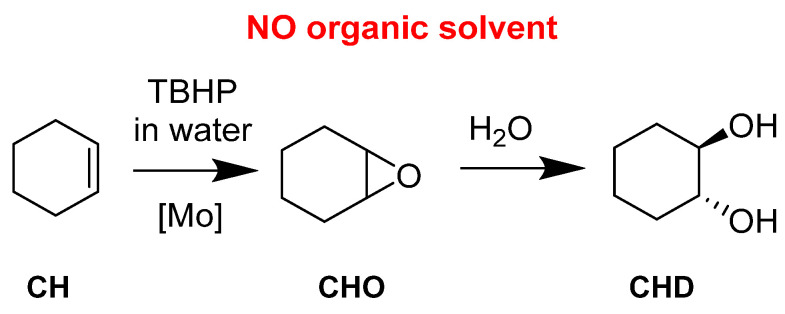
Organic solvent-free cyclohexene epoxidation and subsequent ring-opening with water.

Cyclohexene (CH) has been studied because the corresponding epoxide (CHO) can be readily opened in the presence of water and leads to the *trans*-cyclohexanediol (CHD). (Figure 12) Further steps can lead to the complete opening of the 6-membered ring toward a very valuable species, adipic acid. The activity of the catalyst was also assessed. With a very active catalyst, the ring opening could be quickly observed. It explained why a less active catalyst did not give the diol in big quantities. This was interesting and has been used for the other presented works with applicative purposes.

#### 2.2.2. Application to Biomass Substrates

The success of the process with simple liquid cyclic alkenes was also the basis of extra development toward the valorization of biomass with the epoxidation of a sesquiterpenes [48] and lignans [49], showing that the [MoO_2_(SAP)] coupled with TBHP with/without organic solvent-free conditions could replace the *m*-CPBA method. The species extracted from natural sources are interesting sources of chemicals [50].

*Himachalenes*

**Figure 13:**
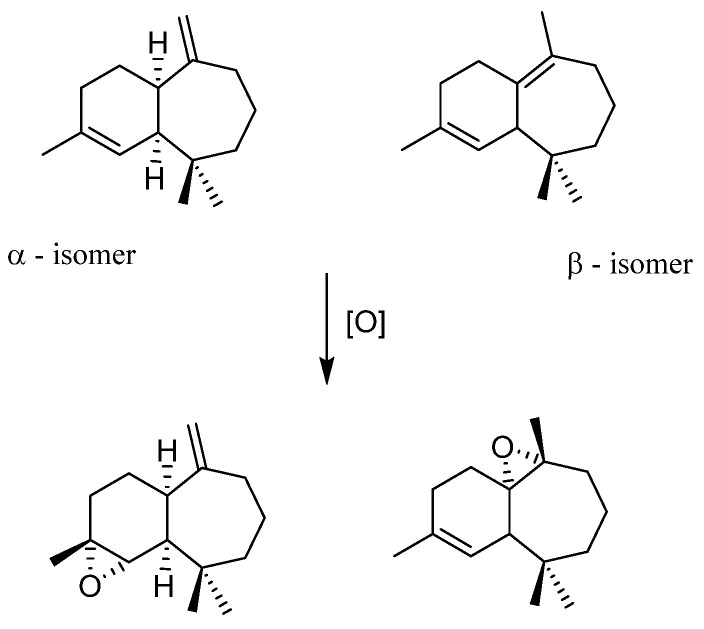
α and β isomers of himachalenes and related epoxides.

Himachalenes are sesquiterpenes obtained from the essential oil of an endemic Moroccan Tree, *Cedrus atlantica* [51]. Those species are relatively abundant and cheap interesting substrates. Those epoxidations exist with classical organic methods, i.e., *m*-CPBA oxidant in CH_2_Cl_2_, a solvent to avoid in industrial or academic processes. *m*-CPBA is known to be efficient but post-treatment is tedious, time-consuming, and contains one step with a basic phase that could be avoided by the use of a more atom-economic oxidant. Thus, in collaboration with a Moroccan research group expert within himachalene chemistry, the epoxidation reaction of three different substrate types was performed. [48]The starting mixture of himachalenes contains the different positions of double bonds present on the seven-membered ring, leading to a mixture of three isomers (Figure 13). It was possible to see that the regioselectivity of epoxidation with MoO_2_(SAP)/TBHP is identical to that using m-CPBA, privileging epoxidation on the internal bonds of the 7-member ring (β isomer) vs. α.

**Figure 14:**
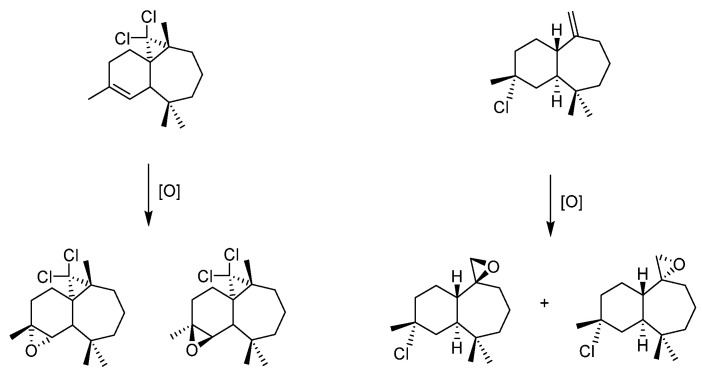
Epoxidation of “protected” himachalenes, leading to addition on the less reactive double bonds when himachalenes are unprotected.

After suppressing one internal double bond through different pathways on the α(β) isomer, only one internal (external) double bond is present to study the diastereoselectivity of the approach and it was here seen that epoxidation could be achieved with stereoselectivity slightly different than the *m*-CPBA method (Figure 14).

*Lignans*

**Figure 15:**
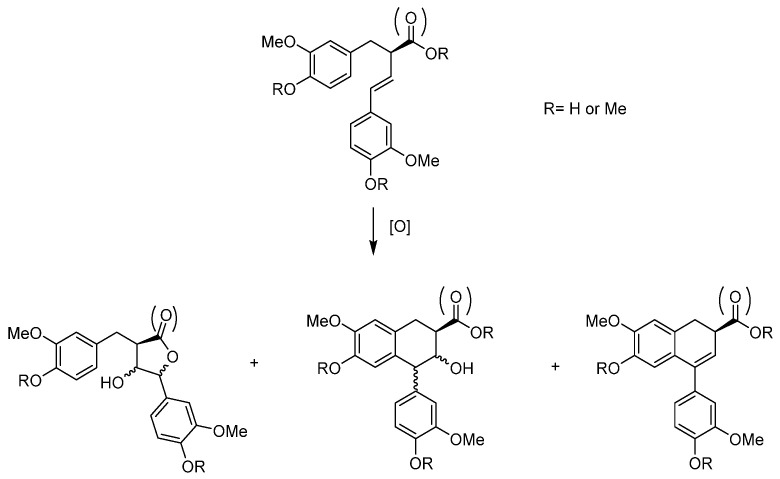
Schematic representation of the oxidation products of 9-norlignans, tetrahydrofuronato-, aryltetralin, and butyrolactones norlignans.

The MoO_2_(SAP)/TBHP catalytic system was tested on lignans [49]. The specific lignans studied-extracted from the knotwood of Norway Spruce (*Picea Abies*)- constitute interesting renewable biphenolic material studied in collaboration with a research group from Abo Akademi in Finland [52,53]. Those compounds, derived from imperanene and imperaneic acid, possess a very interesting double bond between two aromatics. Their oxidation products—through the formation of non-isolable epoxide followed by acid-assisted epoxide ring opening and rearrangements—can lead to different heterocyclic species, such as tetrahydrofuranes, lactones or tetralin structures (Figure 15). The [MoO_2_(SAP)]/TBHP method needs the use of an organic solvent since the substrate was solid. At the difference with the sesquiterpenes, the complexity of the lignans led to oligomeric side products that diminished the yields.

*Limonene*

**Figure 16:**
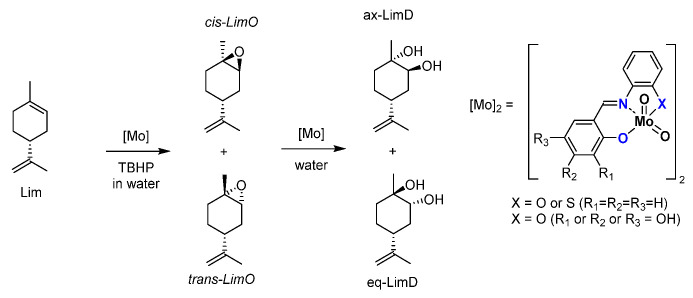
Schematic epoxidation of limonene (Lim) into epoxides (LimO) and water opening with water in limonene diols (LimD).

Another interesting part concerns a very simple substrate, limonene. [54] A byproduct of the orange juice industry, this terpene is cheap and relatively abundant. Used in the food and perfume industries, it can be at the base of building blocks for pharmaceutical compounds. The epoxidation of the limonene (Figure 16) is preferential on the inner double bond and can create two stereoisomers, *cis*- and *trans*- limonene epoxides (LimOs). Those epoxides are useful in different applications, from a molecular point of view, as precursors of several biobased polymers. Both LimOs are relatively stable but the presence of water can open both in two different diols, named here equatorial (*eq*) and axial (*ax*) limonene diols (LimDs). The reactivity favors the stable *ax*-LimD. With the use of MoO_2_L complexes, it has been shown that organic solvent-free conditions led to the formation of both LimOs without difference in selectivity. The interesting point lies in the ring opening of both limOs that showed in major form the *ax*-LimD (exclusively this species starting from *cis*-LimO) but also the formation of the unfavored *eq*-LimD without real caution within the experimental conditions, which is different from all other described protocols for this specific LimD. Indeed, to compare, the existing methods to produce the “unusual” *equ*-LimD requires first the isolation of the *trans*-LimO from a *cis/trans* LimO mixture and the use of mercuric reagent [55] or enzymatic protocols [56] under buffered conditions. An interesting comparison between [MoO_2_(SAP)] and [MoO_2_(SATP)] exhibited that the ONS coordination sphere around the molybdenum favored the *eq*-LimD generation.

*Carveol Oxidation*

**Figure 17:**
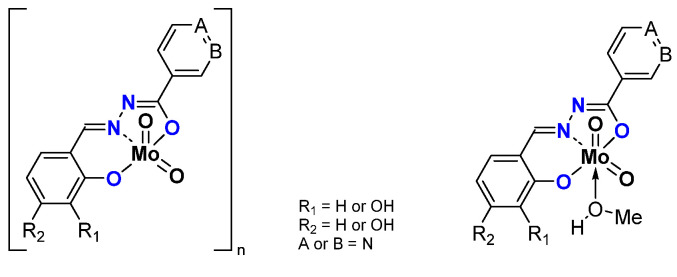
Schematic representation of neutral monomeric (**right**) and polymeric (**left**) Mo species studied containing the salicylaldehydato fragment in the hydrazonato ligand.

**Figure 18:**
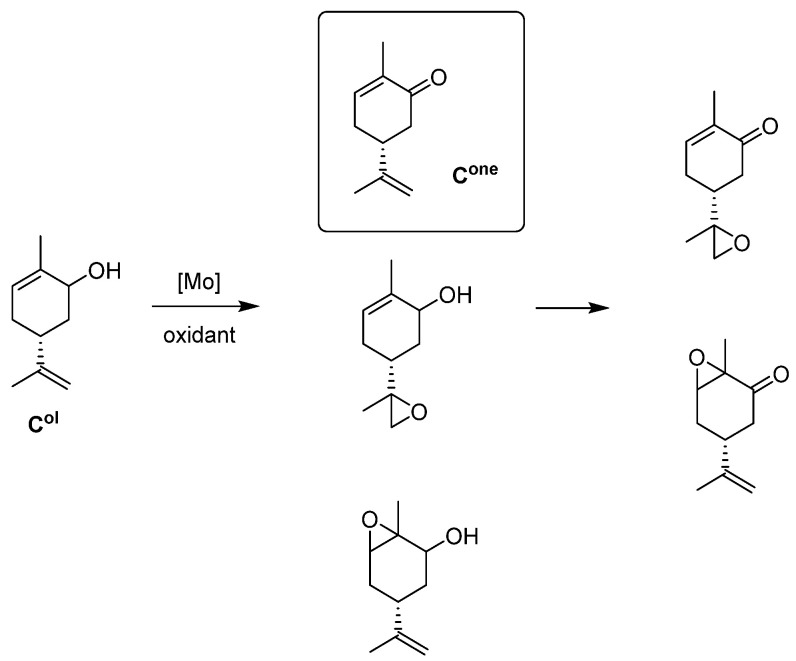
Schematic oxidation of carveol (C^ol^) into carvone (C^one^) and epoxides.

With Mo complexes (Figure 17) similar to those used in Figure 8, oxidation of several alcohols was done (Figure 18). One alcohol of biomass origin, carveol, is the most interesting. The oxidants tested have been H_2_O_2_ and TBHP (water or decane) [57]. Results compiled in Table 9 showed different phenomena. H_2_O_2_ was the best oxidant to transform into carvone (40% selectivity) vs. 4–20% with TBHP. TBHP in decane reacted faster but certainly to the corresponding epoxide. With TBHP, the presence of water seems detrimental for alcohol oxidation. The main factor influencing the activity is the OH on the ligand in position R_1_ that seems to more quickly activate the catalyst. Some further kinetic studies showed that *cis*-carveol is preferentially transformed with H_2_O_2_ and *trans*-carveol with TBHP.

## 3. Vanadium Species

As for molybdenum, vanadium is an element used in oxidation processes. In addition to being present in nature in some enzymatic processes, vanadium can activate smoother and cleaner oxidants, such as TBHP or H_2_O_2_ and the most convenient oxidant, O_2_. Several vanadium-containing compounds have been shown to be active in catalysis, for example the species used by Mimoun [58], Rehder [59], Maurya [60], or Hartung [61]. The mechanisms are numerous according to the nature of the ligands.

### 3.1. SAP

**Figure 19:**
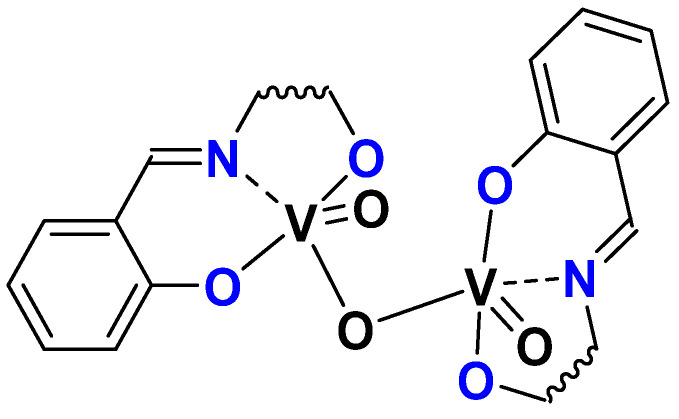
Schematic representation of [VO(L)]_2_O complexes (L = SAP, SAE, SAMP).

It was interesting to take advantage of the H_2_L ligands (H_2_SAE, H_2_SAMP, and H_2_SAP) presented in the molybdenum section and to study the activity of equivalent vanadium species. Through reaction with [VO(acac)_2_] as a vanadium precursor and subsequent oxidation, the dinuclear complexes [(L)VO]_2_O complexes have been isolated (Figure 19) and the structure of the compound (L = SAE, SAMP) was determined through X-ray crystallography. The catalytic activity of 1 mol% complex vs. substrate was tested with [VO(SAP)]_2_O as a catalyst, with both TBHP or H_2_O_2_ as oxidant, and for the first time under organic solvent-free conditions. The epoxidation of cyclo-octene gave a 94% conversion (and 83% selectivity towards the epoxide) after 5.5 h with TBHP and no reaction with H_2_O_2_ [62].

### 3.2. ONO, ONS, and Mechanism

**Figure 20:**
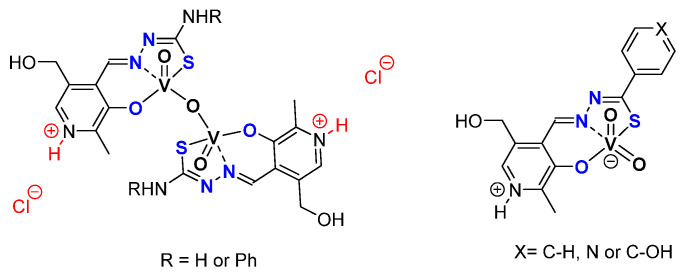
Schematic representation of vanadium complexes containing pyridoxal fragments being monomeric when containing hydrazide moieties and dimeric with thiosemicarbazones.

**Figure 21:**
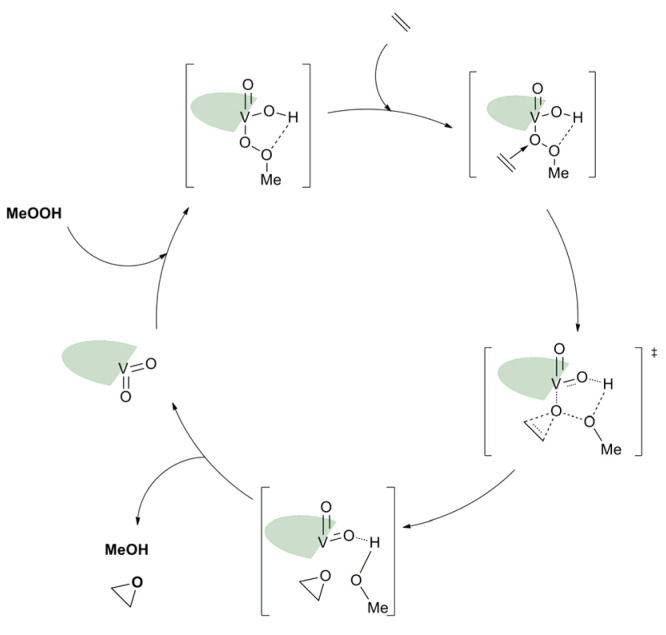
Schematic catalytic cycle proposed after DFT calculations with the [VO_2_(LH)] (X = C-OH) complex. The ligand was schemed with the green symbol.

Based on the pyridoxal moiety used for the Mo complexes mentioned above, different families of vanadium complexes were synthesized with an ONS coordination sphere and containing one vanadium atom with general formulas [VO_2_(LH)] and an ONO coordination sphere around the vanadium creating neutral [V_2_O_3_L_2_] or charged [V_2_O_3_(HL)]Cl_2_ molecules containing two vanadium atoms (Figure 20) [63]. Those species have been tested as catalysts for the epoxidation of cyclo-octene under organic solvent-free conditions (Table 10). With TBHP in water as oxidant. Results were moderate but it was interesting to try to elucidate the mechanism through DFT. Thus, from the complexes of general formulas [VO_2_(LH)] (X = C-OH), calculations showed that the most energetically favorable pathway went through the formation of hydroxido-alkylperoxido [VO(OH)(OOMe)(HL)] (Figure 21) [63].

## 4. Keggin-Type Polyoxometalates as (ep)Oxidation Catalyst

Keggin Polyoxometalates (POMs) is the second class of catalysts studied using the sustainable methods presented herein. Known for a very long time for fundamental research but also for their applications in biology and in catalysis, in both homogeneous and heterogeneous conditions, POMs have the advantage of being an extremely stable species, very simple to be synthesized (mostly in solution methods but recently using solvent-free methods using mechanochemical activation). Those species can very easily activate smooth oxidants (H_2_O_2_, TBHP, O_2_, UHP) for several oxidation reactions. Among the active species relative to POMs; the classical Venturello–Ishii catalyst, a peroxo-oxomolybdenum complex, has been deeply studied and Keggin species were more explored for the sulfoxidation reaction.

### 4.1. Pyridinium Salts

**Figure 22:**
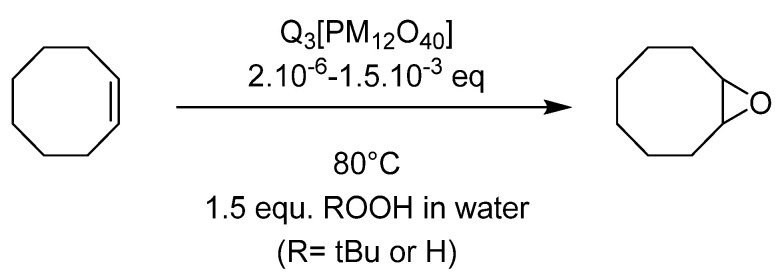
Epoxidation of cyclooctene catalyzed by organic salts of polyoxometalates Q_3_[PM_12_O_40_] (M = Mo or W, Q = Bu_4_N (TBA), Pyr-C_4_H_9_ (BP), Pyr-(CH_2_)_15_CH_3_ (CP).

Very simple [PMo_12_O_40_]^3−^ and [PW_12_O_40_]^3−^ Keggin type heteropolyanions have been tested for catalysts as organic salts using tetrabutylammonium (TBA), butyl- (BP) or cetyl-pyridinium (CP) as cation, in order to use the catalyst in the organic medium, i.e., the substrate itself [64]. The reactions were done without organic solvent and oxidants (H_2_O_2_ or TBHP) in aqueous solution (Figure 22). The results (Table 11) exhibited activity depending on all parameters, i.e., PMo_12_ vs. PW_12_, nature of cation and nature of oxidant. With 0.1% POM vs. cyclo-octene, at 80 °C, TBHP gave a better selectivity toward epoxide without formation of the cyclo-octanediol. This latter compound was observed when H_2_O_2_ was used as oxidant, this certainly explained the low selectivity. The catalysts could be recycled and separated easily from the reaction mixture very easily at room temperature in the case of TBA and BP salts, the CP giving an emulsion that was hard to separate (although efficient). The reaction certainly takes place in the organic phase, and it was found that homogenous and heterogeneous reactions coexist (the solubility of the POMs being strongly cation dependent), explaining the difference within the selectivity. The alkyl pyridinium being the most soluble, a different test with a low catalyst charge was performed, exhibiting activity until 2 or 5 ppm POM vs. substrate ratio but a lower selectivity. Thus, it was supposed that a heterogeneous process gave better selectivity within the reaction media. In addition, it was also shown that [PMo_12_O_40_]^3−^ species were more efficient with TBHP and [PW_12_O_40_]^3−^ with H_2_O_2_.

### 4.2. Supported Catalysts

Within the aim of a sustainable and recyclable process, it was interesting to further study the catalytic activity of the simple Keggin polyanions once ionically grafted on solid support. Two types of supports have rightly been studied: functionalized organic polymer and silica nanoparticles.

#### 4.2.1. Grafted POMs on Merrifield Resins [65]

**Figure 23:**
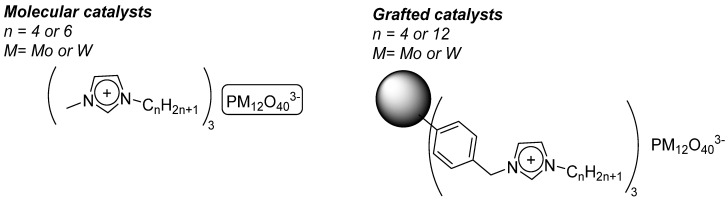
molecular and grafted catalysts on functionalized Merrifield resin.

The protocol consisted in the functionalization of a commercial Merrifield resin by quaternization of alkyl imidazoles by the chloromethyl pending functions present on the polymer. From those functionalizations, [PM_12_O_40_]^3−^ (M= Mo, W) were ionically grafted on those polymers. In order to compare the activity when grafted or as a free molecule, molecular analogs with same type of imidazolium countercations have been also synthesized. (Figure 23) The species were stable and could be characterized through several methods. It was possible to load more Mo Keggin than W Keggin on the Merrifield resins with a range of 55.6–66.7 μmol/g polymer for PMo_12_O_40_ and 12.2–18.9 μmol/g polymer for PW_12_O_40_.

**Figure 24:**
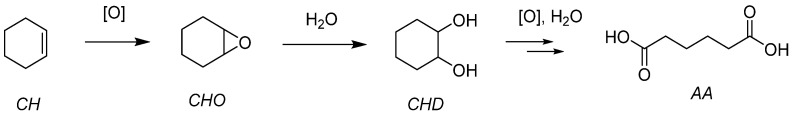
Epoxidation of cyclohexene leading to adipic acid.

The organic and the grafted salts of POMs have been tested for the epoxidation of cyclohexene, an interesting precursor for the synthesis of adipic acid (AA) (Figure 24). With four equivalents of oxidant starting from cyclohexene (Table 12), AA yields from 46–61% were obtained with molecular catalysts and 33–51% with grafted catalysts. The interesting fact lies in the low POM content in general (0.025% POM vs. substrate with molecular catalysts and within 0.001–0.007% range for the grafted catalysts). The study considered each step of the postulated mechanism.

#### 4.2.2. Grafted POMs on Functionalized Silica 

**Figure 25:**
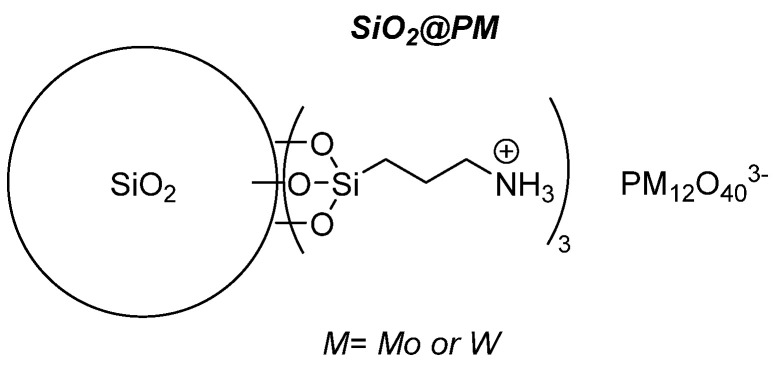
Schematic representation of POMs grafted on functionalized silica.

The ionic grafting of POMs being a convenient recovery method, we continued in this area by using an inorganic support, i.e., ca. 76 nm diameter sized non-mesoporous silica nanoparticles functionalized at their surface by aminopropyltriethoxysilane [66]. The strategy implied a limited number of synthetic steps, ionically grafting the POM at the surface of the functionalized bead through protonation of the pending NH_2_ functions. (Figure 25) Using this synthetic strategy and several characterization methods, the objects contain 0.12–0.14 mmol POM/g of sample, i.e., 2–10 times more than for the Merrifield resin. Those objects were used for the epoxidation of cyclooctene (CO) (Table 13), cyclohexene (CH) (Table 14), and limonene (Lim) (Table 15) and for the oxidation of cyclohexanol (CYol) (Table 16). For CO, the conversion was a bit slower for the grafted catalysts and the selectivity was better for the H_3_PMo_12_O_40_ catalytic objects. The same trend between the metals was observed with CH, the grafted W-catalyst being more active than the heteropolyacids precursors and the reaction giving other products than CHD (maybe AA). With Mo, the CHD was more visible showing that the reaction was slower. With Lim, the reaction was very fast and mainly led to the formation of LimDs, as well as a few quantities of carveol (Col) and carvone (Cone).

CyOH gave interesting information, exhibiting better activity for the heteropolyacids, certainly due to the inner acidity (compared to the grafted ones).

Those objects were even reused and showed recyclability after the third run.

## 5. Conclusions

It has been shown here all the different directions taken in Castres, France toward sustainable processes. Ligand engineering (with some mechanistic DFT explanations) for the coordination complexes and catalysts grafting were the strategies employed to use a very low quantity of catalysts for different (ep)oxidation processes. All has been progressively oriented recently toward the valorization of biomass substrates, in order to situate this research in the context of circular economy. The advances in this research are still in progress.

## Figures and Tables

**Figure 26:**
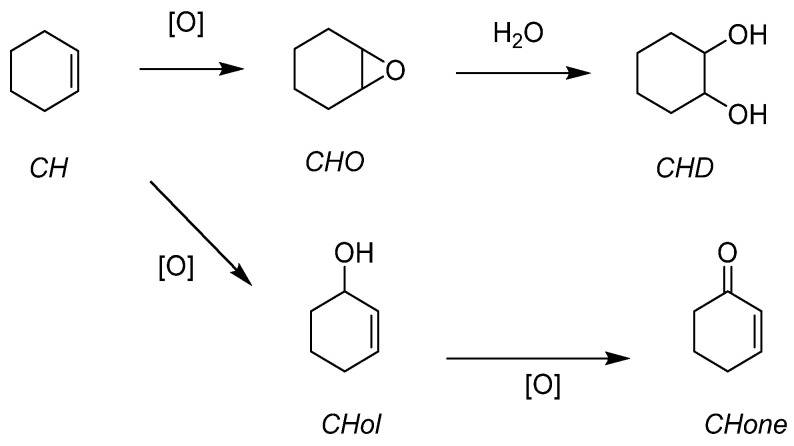
CH epoxidation and studied species.

**Figure 27:**
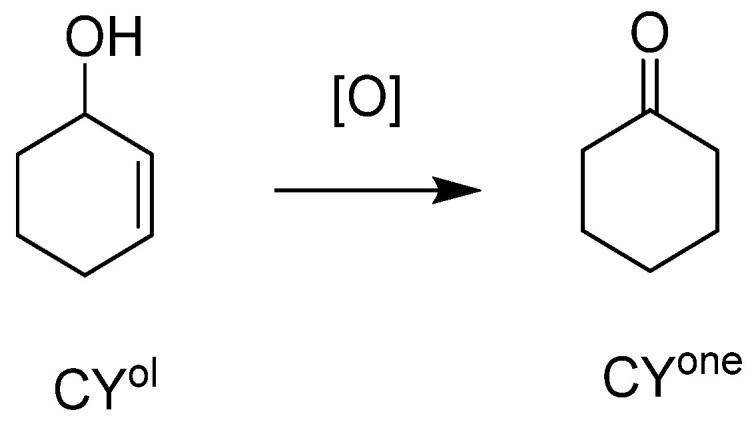
Cyclohexanol (CY^ol^) oxidation and the studied Cyclohexanone (CY^one^) product.

**Table 1:** Results of cyclo-octene (CO) epoxidation under organic solvent-free conditions and selectivity towards cyclo-octene epoxide (COE) after 4 h. Comparison of first and second sphere influence (Structures are those from Figure 4) and corresponding transition state.

General Comparisons	Substitution	Mo (%)	COConv. (%)	COESel. (%)	TS (kcal/mol)
ONX Coordination Sphere	X = O	1.00	64	93	22.5
		0.60	66	90	
		0.33	65	82	
		0.24	63	79	
		0.15	59	74	
		0.10	50	73	
	X = S	1.00	95	89	22.3
		0.60	94	98	
		0.11	86	94	
		0.05	82	94	
		0.025	68	93	
R_2_ (NEt_2_) and/or R_5_ (NO_2_)	-	0.25	71	94	22.5
	R_5_		86	96	21.0
	R_2_		62	93	23.8
	R_5_ + R_2_		73	91	22.3
OH (R_1_, R_2,_ R_3_)	-	0.50	76	93	22.5
	R_1_		92	93	22.4
	R_2_		73	85	22.9
	R_3_		81	91	22.6
OMe (R_1_) and/or Me (R_4_, R_5_)	-	0.50	68	92	22.5
	R_4_		68	91	n.c.
	R_5_		75	91	n.c.
	R_1_		83	94	n.c.
	R_1_ + R_4_		74	94	n.c.
	R_1_ + R_5_		80	92	n.c.

80 °C/4 h/2 eq TBHPaq.

**Table 2:** Results of cyclo-octene (CO) epoxidation and selectivity towards cyclo-octene oxide (COE) under organic solvent free conditions catalyzed with MoO_2_L complexes from Figure 5.

General Formulas	R	CO Conv. (%)	COE Sel. (%)	TOF_20min_	TON
MoO_2_L(MeOH)	Ph	97	97	3360	1940
[MoO_2_L]_n_	Ph	54	84	645	1040
	Me	71	92	960	1420
	H	69	99	1080	1380
[MoO_2_LH(MeOH)]Cl	Ph	48	74	480	960
{[MoO_2_LH]Cl}_n_	Ph	70	82	787	1960
	Me	78	86	1080	1253
	H	79	89	1680	1580

0.05% Mo loading vs. substrate 80 °C/6 h/2 eq TBHPaq.

**Table 3:** Results of cyclooctene (CO) epoxidation and selectivity towards cyclo-octene oxide (COE) with MoO_2_L complexes from Figure 6.

General Formulas	X	CO Conv. (%)	COE Sel. (%)	TOF_20min_	TON
[MoO_2_L(MeOH)]	C-H	41	72	900	200
	N	56	86	1200	1080
	C-OH	67	75	900	1340
[MoO_2_L]_n_	C-H	72	87	484	1440
	N	54	87	818	1120
	C-OH	23	73	940	531
[MoO_2_LH]_2_(Mo_6_O_19_). 2 CH_3_CN	C-H	72	58	44	99
	N	72	53	44	97
	C-OH	76	55	72	105

0.05% Mo loading vs. substrate 80 °C/6 h/2 eq TBHP.

**Table 4:** Results of CO epoxidation and selectivity toward COE under organic solvent free conditions with MoO_2_L complexes from Figure 7.

General Structures	Substitution on				Oxidant TBHP in	CO Conv. (%)	COE Sel. (%)	TOF_20min_	TON
	X	R_1_	R_2_	R_3_					
[MoO_2_L(EtOH)]	C-OH	OMe			Water (W)	89	53	351	368
			OMe			83	50	344	343
						86	46	469	354
[WO_2_L(EtOH)		OMe				17	13	190	70
			OMe			31	9	260	128
[WO_2_L]_n_			-			16	15	169	68
[WO_2_L(MeOH)]	N	OMe			W	17	12	171	70
					W + ACN	51	2		
					Decane (D)	22	12		
			OMe		W	23	8	111	93
					W + ACN	63	2		
					D	25	19		
				OMe	W	22	6	168	90
					W + ACN	35	1		
					D	23	13		
[WO_2_L(EtOH)]		OMe			W	20	3		
					W + ACN	66	1	73	63
					D	20	31		
			OMe		W	28	33		
					W + ACN	78	3	81	117
					D	28	7		
				OMe	W	31	11		
					W + ACN	47	2	204	129
					D	31	19		

0.25% Mo loading vs. substrate 80 °C/6 h/2 eq TBHP.

**Table 5:** Results of CO epoxidation and selectivity towards COE under organic solvent free conditions with MoO_2_L complexes from Figure 8.

General Structures	Substitution on				Oxidant TBHP In	CO Conv. (%)	COE Sel. (%)	TOF_20min_	TON
	A	B	R_1_	R_2_					
[MoO_2_L]_n_					W	36	45	116	151
			OMe			27	56	72	113
				OMe		49	67	119	192
[MoO_2_L]_4_		N		OMe		78	85	151	204
[MoO_2_L]_n_					D	66	78	225	273
			OMe			74	85	200	305
				OMe		79	87	221	324
[MoO_2_L]_4_				OMe		99	94	1152	397
[MoO_2_L]_4_	N				W	79	85	243	309
			OMe			73	93	175	290
				OMe		78	85	171	300
					D	>99	87	153	399
			OMe			>99	92	155	398
				OMe		>99	99	1152	397

Reaction conditions: time, 5 h; temperature, 80 °C. [Mo]/cyclooctene/TBHP molar ratio: 0.25/100/200 for all compounds.

**Table 6:** Results of CO epoxidation under organic solvent free conditions with MoO_2_L complexes of Figure 9.

General Structure	Mo (mol%)	Substitution on				CO Conv. (%)	COE Sel. (%)	TOF_20min_	TON
		X_1_	X_2_	R_1_	R_2_				
[MoO_2_L(MeOH)]	0.25		NH_2_			47	72	84	194
				OMe		56	86	75	229
					OMe	63	85	97	257
[MoO_2_L(EtOH)]						65	82	76	268
				OMe		59	79	95	241
					OMe	85	85	58	350
[MoO_2_L]_2_						38	78	113	160
				OMe		84	90	295	380
					OMe	89	86	298	386
[MoO_2_L(MeOH)]		NH_2_				94	93	702	390
[MoO_2_L(EtOH)]						97	94	642	403
[MoO_2_L]_2_						89	93	345	369
	0.05					83	88	1689	1714
	0.01					82	54	921	2263
	0.25			OMe		90	92	343	372
	0.05					75	85	602	1898
[MoO_2_L(MeOH)]	0.25				OMe	85	90	383	349
[MoO_2_L]_2_						87	91	345	330
	0.05					69	93	954	1301

Reaction conditions: 5 h; 80 °C. [Mo]/CO/TBHP molar ratio: 0.25/100/200.

**Table 7:** Results of CO epoxidation and selectivity toward COE under organic solvent free conditions with MoO_2_L complexes of Figure 10.

General Structures	Substitution on		Oxidant TBHP in	CO Conv. (%)	COE Sel. (%)	TOF_20min_	TON
	X_1_	X_2_					
[MoO_2_L]_n_			W	90	88	290	361
	OMe			92	90	274	368
		OMe		96	93	391	386
[MoO_2_L(MeOH)]				96	95	496	400
	OMe			94	92	319	373
		OMe		99	85	326	351
[MoO_2_L]_n_			D	99	91	290	397
	OMe			98	90	290	397
		OMe		99	90	625	399
[MoO_2_L(MeOH)]				99	93	796	400
	OMe			99	93	298	399
		OMe		99	92	214	400

Reaction conditions: 6 h; 80 °C. [Mo]/CO/TBHP molar ratio: 0.25/100/200.

**Table 8:** Results of CO epoxidation under organic solvent free conditions with MoO_2_L complexes from Figure 11.

	Substitution on								
General Structure	X_1_	X_2_	R_1_	R_2_	Oxidant	COconv. (%)	COE Sel. (%)	TOF_tmin_	TON
[MoO_2_L]_2_	NH_2_		OH		TBHP in W	94	83	370	378
[MoO_2_L(MeOH)]						93	90	406	365
[MoO_2_L]_2_.CH_3_CN		NH_2_				90	82	346	357
[MoO_2_L(MeOH)]						91	87	332	362
[MoO_2_L]_2_.CH_3_CN	NH_2_			OH		89	89	183	358
[MoO_2_L]_2_.CH_3_CN *						86	96	204	339
[MoO_2_L(MeOH)]						83	94	179	360
[MoO_2_L]_2_		NH_2_				58	82	36	234
[MoO_2_L(MeOH)]						50	85	42	270
[MoO_2_L]_2_	NH_2_		OH		TBHP in D	>99	91	1005	400
[MoO_2_L(MeOH)]						>99	92	9415	400
[MoO_2_L]_2_.CH_3_CN		NH_2_				>99	93	1197	400
[MoO_2_L(MeOH)]						>99	89	9556	400
[MoO_2_L]_2_.CH_3_CN	NH_2_			OH		>99	90	8119	367
[MoO_2_L]_2_.CH_3_CN *						>99	95	2445	354
[MoO_2_L]_2_		NH_2_				13	57	106	50
[MoO_2_L]_2_	NH_2_		OH		H_2_O_2_ in W	12	20	13	49
[MoO_2_L(MeOH)]						5	52	22	20
[MoO_2_L]_2_.CH_3_CN		NH_2_				10	26	7	41
[MoO_2_L(MeOH)]						7	49	16	27
[MoO_2_L]_2_.CH_3_CN	NH_2_			OH		16	23	9	62
[MoO_2_L]_2_.CH_3_CN *						15	16	49	61
[MoO_2_L]_2_		NH_2_				16	14	96	63

Reaction conditions: 5 h; 80 °C. [Mo]/CO/TBHP molar ratio: 0.25/100/200. The (*) indicate another polymorph of the complex.

**Table 9:** Results of carveol oxidation and selectivity toward carvone with MoO_2_L complexes from Figure 17.

	Substitution on								
General Structure	R_1_	R_2_	A	B	Oxidant	Conv. (%)	Sel. (%)	TOF_tmin_	TON
[MoO_2_L]_n_	OH		N		H_2_O_2_	88	41	113	308
	OH			N		87	42	294	339
		OH	N			77	40	4	300
		OH		N		85	44	34	329
[MoO_2_L(MeOH)]	OH		N			87	41	23	360
	OH			N		84	41	286	344
		OH	N			89	37	27	367
		OH		H		91	42	20	394
[MoO_2_L]_n_	OH		N		TBHP in water	64	10	237	235
	OH			N		66	10	29	270
[MoO_2_L(MeOH)]	OH		N			62	11	29	270
	OH			N		56	19	185	271
[MoO_2_L(MeOH)]	OH			N	TBHP in decane	99	4	1001	289

Mo loading n(Mo):n(substrate):n(oxidant) = 1:400:800.

**Table 10:** Results of CO epoxidation under organic solvent-free conditions with vanadium complexes from Figure 20.

General Formula	R or X	Conv. (%)	Sel. (%)	TOF_20min_	TON
[V_2_O_3_L_2_]	R = H	61	35	2339	1251
[V_2_O_3_(LH)_2_]Cl_2_	R = H	87	32	2409	1804
[V_2_O_3_L_2_] × 2 MeOH	R = Ph	67	32	1587	1386
[V_2_O_3_(LH)_2_]Cl_2_ × 2 MeOH	R = Ph	74	36	1930	1551
[VO_2_(LH)] × MeOH × H_2_O	X = C-H	23	10	940	532
[VO_2_(LH)] × MeOH × H_2_O	X = C-OH	33	10	1571	700
[VO_2_(LH)]	X = N	31	13	1179	633

**Table 11:** CO epoxidation under organic solvent free conditions with organic salts of POMs from Figure 22. TBHP/cyclooctene = 1.5, 24 h, 80 °C.

General Formulas	Cat (%)	CO Conv. (%)	COE Sel. (%)	TON	TOF_20min_
(BP)_3_[PMo_12_O_40_]	0.099	90.7	71.6	910	470
	0.010	85.2	72.1	8200	2500
	0.005	83.5	75.7	15,000	5600
	0.002	79.4	75.3	40,000	11,000
	0.001	65.3	65.6	53,000	16,000
	0.0005	52.0	44.5	100,000	35,000
(CP)_3_[PMo_12_O_40_]	0.149	77.7	78.0	520	140
	0.080	77.0	78.6	960	180
	0.030	82.0	79.4	2700	340
	0.004	66.7	67.5	15,000	4100
	0.0005	51.2	47.1	100,000	31,000
	0.0002	44.4	53.1	220,000	66,000

**Table 12:** Results of AA formation according to substrate using molecular of grafted POM-based catalysts from Figure 23.

General Structure	Substitution		Cat	H_2_O_2_	Substrate	S Conv.	AA Yield
	M	n	(%)	(equ.)	S	(%)	(%)
Molecular	Mo	4	0.025	4	CH	65	46
catalysts				2	CHO	>99	36
				2	CHD	>99	74
	W	4	0.025	4	CH	75	61
				2	CHO	>99	47
				2	CHD	>99	72
	Mo	6	0.025	4	CH	61	42
				2	CHO	>99	32
				2	CHD	>99	58
	W	6	0.025	4	CH	68	50
				2	CHO	>99	43
				2	CHD	>99	63
Grafted	Mo	4	0.004	4	CH	58	33
catalysts				2	CHO	>99	28
				2	CHD	71	46
	W	4	0.007	4	CH	61	43
				2	CHO	>99	33
				2	CHD	94	60
	Mo	12	0.003	4	CH	56	30
				2	CHO	>99	31
				2	CHD	79	41
	W	12	0.001	4	CH	73	51
				2	CHO	>99	36
				2	CHD	95	56

**Table 13:** CO epoxidation into COE with heteropolyacids and corresponding anionic species from Figure 25.

Catalyst	Run	Cat x	Conv (%)	Sel (%)	TON
H_3_PW_12_O_40_	1	0.070	64	14	807
SiO_2_@PW	1	0.070	72	41	981
	2		75	38	987
	3		77	37	968
H_3_PMo_12_O_40_	1	0.058	99	44	1712
SiO_2_@PMo	1	0.058	98	71	1693
	2		96	72	1620
	3		93	69	1598

T = 80 °C, t = 24 h, POM/TBHP/CO = x/150/100.

**Table 14:** CH epoxidation with heteropolyacids and corresponding anionic species from Figure 25. Analyzed species are in Figure 26.

Catalyst	Run	Cat x	Conv	Selectivity (%)				TON
			CH	CHO	CHD	CHol	CHone	
H_3_PW_12_O_40_	1	0.014	31	<1	4	3	3	11,307
SiO_2_@PW	1	0.014	45	1	2	4	5	21,458
	2		43	1	2	2	3	20,649
	3		26	3	3	7	7	12,373
H_3_PMo_12_O_40_	1	0.012	91	<1	40	3	2	52,728
SiO_2_@PMo	1	0.012	80	13	26	5	2	46,732
	2		74	15	22	6	3	42,487
	3		60	26	20	5	2	36,345

T = 80 °C, t = 48 h, POM/TBHP/CH = x/150/100.

**Table 15:** Lim epoxidation with heteropolyacids and corresponding anionic species from Figure 24. Analyzed species can be found in Figure 16 and Figure 18.

			Conv	Selectivity (%)						TON
Catalyst	Run	Cat x	Lim	LimO		LimD		C^ol^	C^one^	
				cis	trans	Ax	eq			
H_3_PW_12_O_40_	1	0.070	67	0	0	5	3	1	4	1287
SiO_2_@PW	1	0.070	58	0	3	13	1	8	8	754
	2		59	0	3	13	1	10	8	768
	3		62	0	2	12	2	11	8	754
H_3_PMo_12_O_40_	1	0.058	99	0	0	18	10	1	2	1859
SiO_2_@PMo	1	0.058	91	0	0	36	11	4	3	1721
	2		86	0	0	32	8	6	7	1626
	3		81	0	<1	20	5	6	6	1526

T = 80 °C, t = 24 h, POM/TBHP/CH = x/150/100.

**Table 16:** CY^ol^ oxidation toward CY^one^ with heteropolyacids and corresponding anionic species grafted on silica schemed in Figure 22. Studied products are those from Figure 27.

Catalyst	Run	Cat x	Conv	Sel	TON
			CY^ol^	CY^one^	
H_3_PW_12_O_40_	1	0.070	44	34	525
SiO_2_@PW	1	0.070	11	51	137
	2		8	97	101
	3		7	87	92
H_3_PMo_12_O_40_	1	0.058	58	54	728
SiO_2_@PMo	1	0.058	18	76	228
	2		17	90	207
	3		20	75	249

T = 80 °C, t = 24 h, POM/TBHP/CH = x/150/100.
